# Supplementary material for: TBX3 acts as tissue-specific component of the Wnt/β-catenin transcriptional complex
Source: eLife. 2020 Aug 18;9:e58123. doi: 10.7554/eLife.58123 (PMC7434441; doi:10.7554/eLife.58123)
Supplement: Supplementary file 2. [file elife-58123-supp2.docx]

Primers used in ChIP-qPCR

All primers are indicated in the 5´>3´ direction

*AXIN2* promoter:

F CTGGAGCCGGCTGCGCTTTGATAA

R CGGCCCCGAAATCCATCGCTCTGA

*AXIN2* enhancer:

F GACGGAGCATTCCAGTGTTT

R CCAGAAGGGACTTCAAAGCA

*AXIN2* negative control region:

F CTGGCTTTGGTGAACTGTTG

R AGTTGCTCACAGCCAAGACA

Primers used in real time, quantitative RT-PCR

*AXIN2*:

F GCAGGCTAGCTGAGGTGTCG

R GGCTCCCGTCTGAACAGTGG

*GAPDH*:

F CAATGACCCCTTCATTGACC

R GACAAGCTTCCCGTTCTCAG

*NKD1*:

F GAAACTTCACTCCAAGCCGG

R CTGTCTCCCGATCCACTCC

*TBX3*:

F AGATGGTCATTACCAAGTCG

R CATCAGCAGCTATAATGTCC
