# Supplementary material for: TBX3 acts as tissue-specific component of the Wnt/β-catenin transcriptional complex
Source: eLife. 2020 Aug 18;9:e58123. doi: 10.7554/eLife.58123 (PMC7434441; doi:10.7554/eLife.58123)
Supplement: Transparent reporting form [file elife-58123-transrepform.pdf]

eLIFE
1st Floor
24 Hills Road
Cambridge CB2 1JP, UK
P 01223 855340
W elifesciences.org
T @elife

# *eLife's* transparent reporting form

We encourage authors to provide detailed information *within their submission* to facilitate the interpretation and replication of experiments. Authors can upload supporting documentation to indicate the use of appropriate reporting guidelines for health-related research (see EQUATOR Network), life science research (see the BioSharing Information Resource), or the ARRIVE guidelines for reporting work involving animal research. Where applicable, authors should refer to any relevant reporting standards documents in this form.

If you have any questions, please consult our Journal Policies and/or contact us: editorial@elifesciences.org.

**Sample-size estimation**

- You should state whether an appropriate sample size was computed when the study was being designed
- You should state the statistical method of sample size computation and any required assumptions
- If no explicit power analysis was used, you should describe how you decided what sample (replicate) size (number) to use

Please outline where this information can be found within the submission (e.g., sections or figure legends), or explain why this information doesn't apply to your submission:

Each of our experiments was conducted at least three times. No use of power analysis to compute the appropriate sample size was used. When applicable, statistical analyses and the test used are reported during the description of the result and within the figure legends.

**Replicates**

- You should report how often each experiment was performed
- You should include a definition of biological versus technical replication
- The data obtained should be provided and sufficient information should be provided to indicate the number of independent biological and/or technical replicates
- If you encountered any outliers, you should describe how these were handled
- Criteria for exclusion/inclusion of data should be clearly stated
- High-throughput sequence data should be uploaded before submission, with a private link for reviewers provided (these are available from both GEO and ArrayExpress)

Please outline where this information can be found within the submission (e.g., sections or figure legends), or explain why this information doesn't apply to your submission:

Each of our experiments was conducted at least three times, with the exception of ChIP-seq (Figure 3) for which we did two independent replicates. In each experiment we indicate the number of replicates within the figure legend describing it (as in "N=30", figure legend 1D). We considered biological replicates as experimental results obtained on different mouse individual (such as those represented in Figures 1C, 1D, 1E) or embryos (such those in Figure 4A, B). When using cultured cells, biological replicates were considered as experiments done in different moments, with the use of re-made batches of each reagent used (including the cultured cells; e.g. Figure 4C). In one case we represent a technical replicate as result of different qPCR-based measurements on the same template DNA (e.g. Figure 4D). Note that this is intrinsically a replication of the highly controlled experiment shown in Figure 3 (more specifically, see *Axin2* promoter in Figure 3F).
Data deposition: ChIP-seq data have been deposited at ArrayExpress with accession number E-MTAB-8997. The RNA-seq experiment has been deposited at ArrayExpress with accession number E-MTAB-9000. Proteomics Data are available via ProteomeXchange with identifier PXD018805.

**Statistical reporting**

- Statistical analysis methods should be described and justified
- Raw data should be presented in figures whenever informative to do so (typically when N per group is less than 10)
- For each experiment, you should identify the statistical tests used, exact values of N, definitions of center, methods of multiple test correction, and dispersion and precision measures (e.g., mean, median, SD, SEM, confidence intervals; and, for the major substantive results, a measure of effect size (e.g., Pearson's r, Cohen's d)
- Report exact p-values wherever possible alongside the summary statistics and 95% confidence intervals. These should be reported for all key questions and not only when the p-value is less than 0.05.

Please outline where this information can be found within the submission (e.g., sections or figure legends), or explain why this information doesn't apply to your submission:

| |
|---|
| For each experiment we indicate, within the figure legends, the statistical test used as well as the resulting statistical confidence. The obtained p-values are indicated within the respective figure panels to which they are referred. |

(For large datasets, or papers with a very large number of statistical tests, you may upload a single table file with tests, Ns, etc., with reference to sections in the manuscript.)

**Group allocation**

- Indicate how samples were allocated into experimental groups (in the case of clinical studies, please specify allocation to treatment method); if randomization was used, please also state if restricted randomization was applied
- Indicate if masking was used during group allocation, data collection and/or data analysis

Please outline where this information can be found within the submission (e.g., sections or figure legends), or explain why this information doesn't apply to your submission:

| |
|---|
| A case of "blind" count is applicable to the experiment of Zebrafish embryos injection shown in Figure 4E-G. Two groups of embryos were injected with TBX3-OE or CTLR cells. Upon experiment completion, one researcher collected pictures of embryos from the two groups, labelling them in an arbitrary way. Another research would then count the number of observed metastases throughout the pictures without knowing to which group they belonged. The first researcher could then count how many metastases were counted in a blind, unbiased way for each of the two experimental groups. |

**Additional data files ("source data")**

- We encourage you to upload relevant additional data files, such as numerical data that are represented as a graph in a figure, or as a summary table
- Where provided, these should be in the most useful format, and they can be uploaded as "Source data" files linked to a main figure or table
- Include model definition files including the full list of parameters used
- Include code used for data analysis (e.g., R, MatLab)
- Avoid stating that data files are "available upon request"

Please indicate the figures or tables for which source data files have been provided:

eLife Sciences Publications, Ltd is a limited liability non-profit non-stock corporation incorporated in the State of Delaware, USA, with company number 5030732, and is registered in the UK with company number FC030576 and branch number BR015634 at the address 1st Floor, 24 Hills Road, Cambridge CB2 1JP | August 2014

We include a supplemental file that lists the codes and software used in the bioinformatics analyses shown in Figure 3 and Figure 4A-B.
